# H19 lncRNA alters methylation and expression of *Hnf4α* in the liver of metformin-exposed fetuses

**DOI:** 10.1038/cddis.2017.392

**Published:** 2017-12-07

**Authors:** Jie Deng, Martin Mueller, Tingting Geng, Yuanyuan Shen, Ya Liu, Peng Hou, Ramanaiah Ramillapalli, Hugh S Taylor, Michael Paidas, Yingqun Huang

**Affiliations:** 1 Department of Obstetrics, Gynecology, & Reproductive Sciences, Yale University School of Medicine, New Haven, CT 06510, USA; 2 Department of Obstetrics and Gynecology, University Hospital, Bern, Switzerland; 3 Department of Endocrinology, First Affiliated Hospital of Xi’an Jiaotong University School of Medicine, Xi’an 710061, China; 4 Department of Endocrine and Breast Surgery, The First Affiliated Hospital of Chongqing Medical University, Chongqing 400016, China; 5 Department of Veterinary Medicine, College of Animal Science and Technology, Anhui Agricultural University, Anhui 230036, China

## Abstract

Metformin is the most widely used anti-diabetic medication worldwide. However, human and animal studies suggest that prenatal metformin exposure may increase the risk of metabolic disorders in adult offspring, yet the underpinning mechanism remains unclear. Here we report that metformin-exposed mouse fetuses exhibit elevated expression of the H19 long noncoding RNA, which induces hypomethylation and increased expression of hepatocyte nuclear factor 4*α* (HNF4*α*). As a transcription factor essential for morphological and functional differentiation of hepatocytes, HNF4*α* also has an indispensable role in the regulation of expression of gluconeogenic genes. Consistently, H19 overexpression in a human liver cell line leads to decreased methylation and increased expression of *Hnf4α*, with concomitant activation of the gluconeogenic program. Mechanistically, we show that the methylation change of *Hnf4α* is induced by H19-mediated regulation of *S*-adenosylhomocysteine hydrolase. We also provide evidence that altered H19 expression is a direct effect of metformin in the fetal liver. Our results suggest that metformin from the mother can directly act upon the fetal liver to modify *Hnf4α* expression, a key factor for both liver development and function, and that perturbation of this H19/Hnf4*α*-mediated pathway may contribute to the fetal origin of adult metabolic abnormalities.

A hallmark of type 2 diabetes (T2D) and gestational diabetes mellitus (GDM) is hyperglycemia and insulin resistance. Polycystic ovary syndrome (PCOS) is another common endocrine disorder among women of reproductive age. PCOS causes anovulation and infertility, and is often associated with obesity and insulin resistance. Pregnant women with PCOS, GDM, or nondiabetic obesity have a significantly higher risk of adverse pregnancy and infant outcomes, including miscarriages, preeclampsia, fetal macrosomia, preterm birth, and high infant morbidity and mortality.^1^ Metformin is an oral biguanide agent that has been widely prescribed for the treatment of T2D. In the past decade, metformin has been used extensively in the treatment of PCOS and GDM,^1^ and recently in nondiabetic obese pregnant women.^2, 3^ Metformin freely crosses the placental barrier, reaching fetal concentrations of more than 50% of those detected in the maternal circulation.^4, 5^ Therefore, metformin administration during pregnancy has the potential of both direct and indirect (through altering the maternal environment) impact on the fetus.

Interestingly, in a follow-up study of a randomized, controlled trial on PCOS women, the authors reported significantly higher fasting glucose in 8-year-old children born to women treated with metformin compared to placebo from first trimester until delivery.^6^ Likewise, when female mice on regular diet were exposed to metformin throughout pregnancy (despite no significant change in bodyweight), the offspring (both male and female) had a decreased bodyweight at embryonic day 18.5 (E18.5) but gained more weight and mesenteric fat in adult when challenged with high-fat diets (HFD). Notably, the offspring also had enlarged livers despite little changes in the lipid profile. Moreover, the male adult offspring additionally developed elevated fasting glucose and glucose intolerance.^5^ While the molecular mechanism underlying these observations remains to be investigated, the results raise the concern that *in utero* metformin exposure may alter fetal liver development and function, setting the stage for long-term consequences on metabolism in adult life.

One well-studied example of prenatal events that lead to long-term metabolic problems is dexamethasone exposure during the last week of gestation, which causes low birthweight and hyperglycemia and glucose intolerance in adult rats^7^ and sheep.^8^ In rats, a positive correlation between dexamethasone exposure and an elevated expression of hepatocyte nuclear factor 4*α* (HNF4*α*), together with its regulated phosphoenolpyruvate carboxykinase 2 (PEPCK, encoded by *pck1/pck2*) was identified in the fetal liver.^9^ *Hnf4α* encodes a transcription factor essential for hepatocyte differentiation and maturation, both morphologically and functionally. It also acts to regulate expression of key gluconeogenic genes, including *Pck1* and glucose-6-phosphatase (*G6pc*).^10, 11^ Both *Pck1* and *G6pc* encode rate-controlling enzymes that catalyze the first and last steps of gluconeogenesis reaction, respectively.^12^ HNF4*α* binds to well-characterized *cis*-elements on the promoters of *Pck1* and *G6pc*, thereby activating transcription.^10, 13^ As hepatic gluconeogenesis normally does not occur at a considerable level until after birth, the apparently premature activation of gluconeogenesis program may contribute to the adverse consequences on glucose metabolism in adulthood.

The developmentally regulated imprinted *H19*, together with its co-regulated *Igf2*, has an important role in embryo development and growth control. *H19* is abundantly expressed in the placenta and fetal tissues and strongly downregulated postnatally, except in a few adult tissues, including skeletal muscle and heart.^14^ *H19* encodes a multi-functional long noncoding RNA (herein called H19) and has been shown to influence DNA methylation in a genome-wide manner through interaction with *S*-adenosylhomocysteine hydrolase (SAHH).^15, 16^ SAHH is the only eukaryotic enzyme that hydrolyzes *S*-adenosylhomocysteine (SAH), a potent product inhibitor of SAM-dependent methyltransferases, including DNA methyltransferases. H19 binds to SAHH and inhibits its enzymatic activity. This causes accumulation of SAH and inhibition of DNA methyltransferases, leading to decreased DNA methylation.^15, 16^ H19 expression is normally negligible in adult liver, but an elevated expression was noted in human patients with T2D.^17^ Given that excessive hepatic gluconeogenesis contributes significantly to the hyperglycemia in T2D,^12^ we hypothesized that H19 may have a role in gluconeogenesis regulation.

In the present work, we identify altered expression of *Hnf4α* in the liver of prenatally metformin-exposed fetuses. We provide evidence that metformin from the mother can directly act upon the fetal liver to upregulate H19, which in turn induces changes in *Hnf4α* expression via an epigenetic mechanism. Given that HNF4*α* is an essential factor both for liver development and function, our results provide a potential mechanistic insight linking prenatal metformin exposure to adult metabolic disorders.

## Results

### Metformin-exposed fetuses have a decreased bodyweight

Pregnant mice on regular diet were given metformin (or vehicle) orally at a daily dose of 250 mg/kg from embryonic day 0.5 (E0.5) through E18. Fetuses were collected at E18.5. The metformin dosage was chosen based on published studies from the mouse.^5, 18, 19^ This dosage was well tolerated, elicited no recognizable toxicity, and had no significant effects on maternal bodyweight and litter size across different mouse strains (including CD-1 used in the current study).^5, 18, 19^ Further, this dosage was equivalent to that used in the studies of pregnant women with PCOS and nondiabetic obesity.^2, 3, 6, 20, 21^ It produced metformin concentrations in the maternal and fetal circulation in mice similar to those measured in PCOS women.^5, 21^

**Figure 1:**
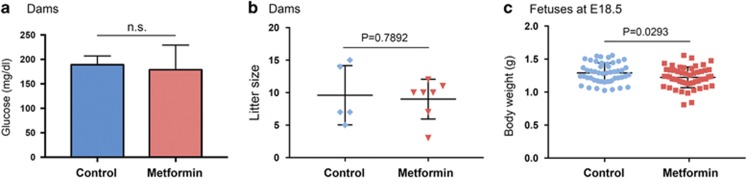
Gestational metformin exposure reduces fetal bodyweight. (**a**) Maternal blood glucose on gestational day 18.5 showing no significant difference between the groups. Control group, *n*(dams)=5; metformin group, *n*(dams)=7. NS, not statistically significant. (**b**) Scatter plots of litter size, with each dot representing a dam. The horizontal line depicts group median and the whiskers mark the interquartile range. (**c**) Scatter plots of fetal bodyweight at E18.5, with each dot represents a fetus. Control group, *n* (fetuses from 5 dams)=48; metformin group, *n*(fetuses from 7 dams)=63. *P*-value indicates statistically significant

Metformin treatment did not alter maternal blood glucose levels (Figure 1a), consistent with previous reports showing no blood glucose-lowering effect of metformin on regular-diet-fed, nondiabetic pregnant mice.^19^ Nor were there significant changes in the litter size (Figure 1b) and viability (data not shown). However, metformin-treated dams produced fetuses with a slightly but significantly lower bodyweight at E18.5 (Figure 1c). This is in line with a published report that gestational metformin-exposed mouse fetuses had a reduced bodyweight at E18.5.^5^ Notably, new born rats prenatally exposed to dexamethasone had a decreased birthweight and also developed hyperglycemia and glucose intolerance in adulthood.^7^ In humans, low birthweight (including those within the normal range, not just severely underweighted babies) is a strong predictor of insulin resistance and diabetes in adult life.^22, 23 24^ Together, our results suggest that low bodyweight may predispose metformin-exposed fetuses to developing metabolic abnormalities in adult life.

### Metformin-exposed fetuses display altered hepatic gene expression

**Figure 2:**
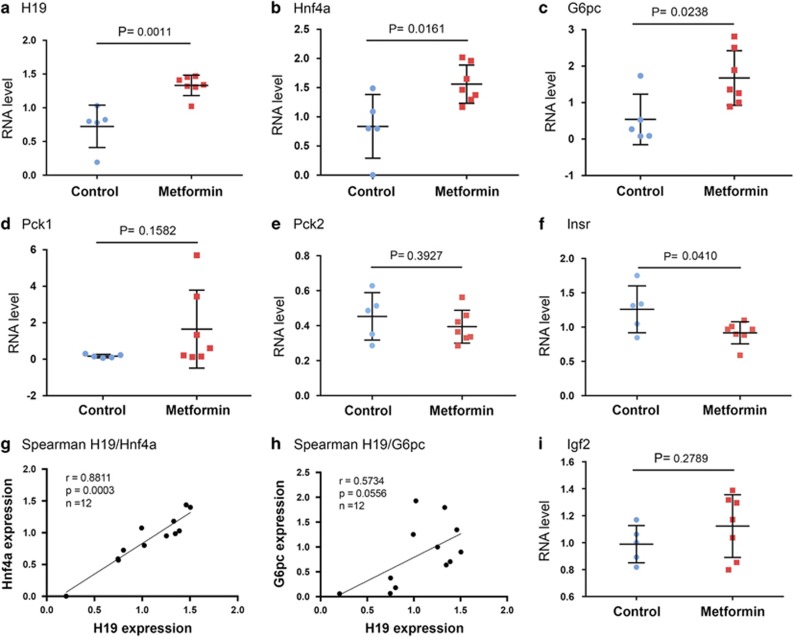
Gene expression in fetal livers. (**a**–**f and** **i**) Scatter plots of RNA levels assessed by RT-qPCR. Control group, *n*(fetuses from 5 dams)=5; metformin group, *n*(fetuses from 7 dams)=7. *P*-values are indicated. (**g** and **h**) Spearman correlation suggests an *in vivo* positive relationship between the expressions of *H19*, *Hnf4α*, and *G6pc*. Spearman correlation coefficient, *P*-value, and sample numbers are marked on the left top of the plot

Evidence from both human and animal studies suggests that gestational metformin exposure has a deleterious effect on glucose metabolism in adulthood.^5, 6^ To begin to elucidate the molecular mechanism that might link prenatal metformin exposure to adult metabolic defects, we examined expression of a subset of genes in control and metformin-exposed fetal livers. The control and metformin group consisted of five and seven pregnant dams, respectively. Livers from one fetus per dam, selected at random, were used for analysis. RNAs were isolated from the livers and gene expression was determined by reverse transcription and quantitative real-time PCR (RT-qPCR) analysis. The metformin-exposed fetuses had a significantly increased expression of *H19*, *Hnf4α*, and *G6pc*, compared to the control group (Figures 2a–c), while the expression of both *Pck1* and *Pck2* were unaltered (Figures 2d and e). Interestingly, the insulin receptor INSR was downregulated in the metformin group (Figure 2f). Importantly, Spearman correlation revealed a strong positive relationship between *H19* and *Hnf4α* (*P*=0.0003; Figure 2g), suggesting an *in vivo* functional interplay between the two genes (see below). There was also a borderline significant correlation between H19 and *G6pc* (*P*=0.0556; Figure 2h). This was not surprising given that *G6pc* is further downstream of the regulatory axis as compared to *Hnf4α*.^10, 11^ There was no significant difference in the expression of *Igf2* between the two groups (Figure 2i), despite that *H19* and *Igf2* were previously reported to be co-regulated in mouse skeletal muscle during development.^14^ Thus, gestational metformin exposure causes altered hepatic expression of *H19* and *Hnf4α*, together with its regulated key gluconeogenic gene *G6pc*, as well as insulin signaling pathway gene *Insr* in the fetus.

### H19 upregulates Hnf4*α* expression and glucose production in hepatic cells

**Figure 3:**
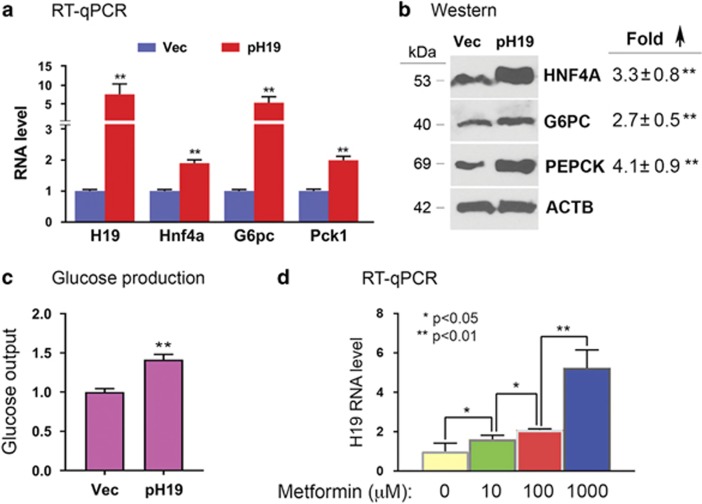
H19 overexpression and metformin treatment in Huh7 cells. (**a** and **b**) Huh7 cells were transfected with Vec or pH19. RNA (**a**) and protein (**b**) were isolated at 24 and 48 h post transfection, respectively, and levels were determined. In (**b**), representative gel images from three independent western blot experiments are shown, with fold increase in pH19-transfected compared to Vec-transfected cells marked on the right. Numbers are mean±S.D. (*n*=3). **P<0.01. The molecular weights of the proteins are indicated on the left. (**c**) Huh7 cells were transfected with Vec or pH19. Glucose output assays were carried out at 48 h post transfection. Numbers are mean±S.D. (*n*=3). **P<0.01. (**d**) Huh7 cells were treated with metformin at the indicated concentrations for 48 h. RT-qPCR results show increased expression of H19 in a dose-dependent manner. Numbers are mean±S.D. (*n*=3). *P-*values are indicated

In light of a previous report on H19 increase in T2D patient livers^17^ and our own observation from human endometrial cancer cells that downregulation of H19 correlated with increased promoter methylation of *Hnf4α* (Supplementary Data S1 in ref. 16), we wanted to test whether H19 might regulate *Hnf4α* in the fetal liver. *H19* expression is robust in fetal hepatocytes but is sharply repressed in fully differentiated hepatocytes after birth.^25^ However, *H19* expression is reactivated in hepatocellular carcinoma.^26^ Thus, we used Huh7 human hepatocellular carcinoma cells as an *in vitro* model system for our studies, because they mimic fetal hepatocytes in terms of *H19* expression. To address whether increased *H19* expression would upregulate *Hnf4α*, Huh7 cells were transfected with a human H19 expression plasmid (pH19)^27^ or control empty vector (Vec), followed by RNA and protein analysis. H19 overexpression increased expression of *Hnf4α*, together with its regulated genes *G6pc* and *Pck1* at both the mRNA (Figure 3a) and protein (Figure 3b) levels. H19 overexpression also enhanced glucose production (Figure 3c). These results suggest that H19 has a causative role in increasing *Hnf4α* expression and gluconeogenesis in hepatic cells.

### Metformin upregulates H19 in hepatic cells

As metformin can cross the placenta to reach the fetus, gestational metformin exposure could potentially affect gene expression of the pregnant dams, the placenta, and the fetus. Thus, the mechanisms underlying gene expression changes in the fetus are likely complex, reflecting both direct and indirect (through modification of maternal and placental functions) effects of metformin. To determine whether the altered expression of H19 might be a direct impact of metformin on the fetal liver, we incubated Huh7 cells with metformin and found that it increased H19 expression in a dose-dependent manner (Figure 3d). Importantly, the metformin doses used were within the therapeutic concentration range found in the plasma of T2D human patients.^28^ Taken together, we propose that the upregulation of H19 in the fetal liver is likely a direct effect of metformin on the fetus.

### *Hnf4α* is hypomethylated in liver of metformin-exposed fetuses

**Figure 4:**
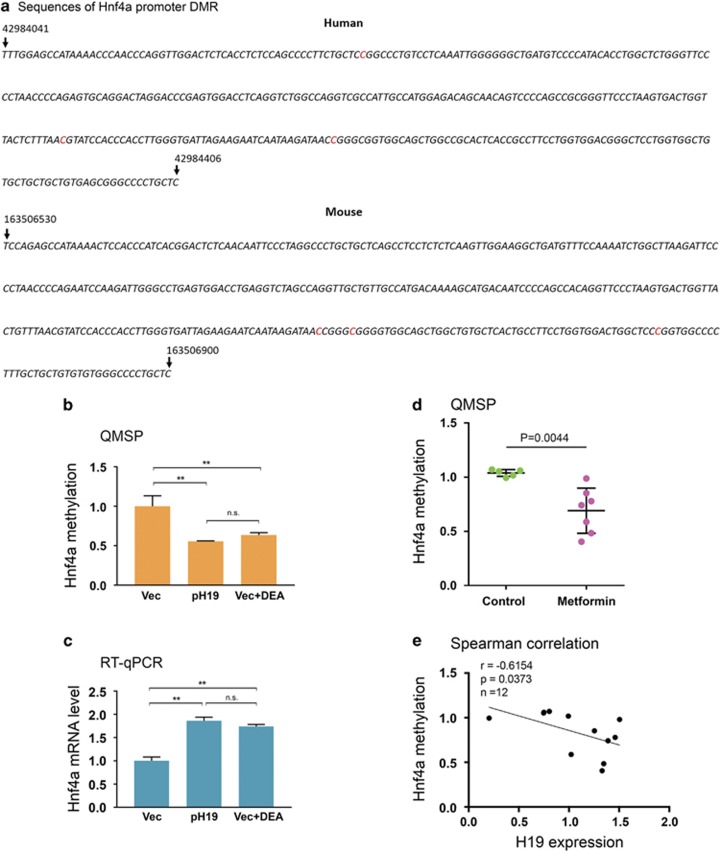
H19 regulates *Hnf4α* methylation *in vitro* and *in vivo*. (**a**) Sequences of DMRs in the conserved promoter region of human and mouse *Hnf4α*. The three differentially methylated cytosine residues are highlighted in red. The numbers on top of the sequences mark the positions of the indicated nucleotides in the chromosomes. (**b**) Huh7 cells were transfected with Vec, pH19, or Vec plus DEA. Genomic DNAs were extracted 15 h later and analyzed by QMSP. Numbers are mean±S.D. (*n*=3). **P<0.01. NS, not statistically significant. (**c**) Huh7 cells were treated as described in **b**. RNAs were extracted 24 h later and analyzed by RT-qPCR. Numbers are mean±S.D. (*n*=3). **P<0.01. NS, not statistically significant. (**d**) Scatter plot of *Hnf4α* methylation in mouse fetal livers. Control group, *n* (fetuses from 5 dams)=5; metformin group, *n*(fetuses from 7 dams)=7. (**e**) Spearman correlation between H19 RNA level and *Hnf4α* promoter methylation, showing a negative correlation

Our previous genome-scale methylation studies from human endometrial cancer cells revealed a correlation between decreased H19 expression and increased methylation at multiple CpG sites within a highly conserved promoter region of human *Hnf4α* (Figure 4a) (Supplementary Data S1 in ref. 16). This suggested to us that H19 may regulate *Hnf4α* expression by affecting methylation of this differentially methylated region (DMR), given that H19 acts to alter DNA methylation genome-wide through interaction with SAHH.^15^ To test this possibility, we transfected Huh7 cells with pH19 or Vec and asked whether increased H19 expression would lead to hypomethylation of the DMR. We compared methylation between control and H19-overexpressed cells by quantitative methylation-specific PCR (QMSP) using a well-established method.^15^ We observed decreased methylation of the DMR in H19-overexpressed *versus* control cells (Figure 4b, compare second column to first column on the left). As H19 acts to inactivate SAHH, thereby decreasing gene methylation,^15, 16^ incubation of Huh7 cells with d-Eritadenine (DEA), a pharmacological inhibitor of SAHH, produced an effect similar to that of H19 overexpression (Figure 4b, compare third column to first column). This suggests that H19 regulates *Hnf4α* promoter methylation via the previously identified H19/SAHH pathway. As decreased promoter methylation in general activates gene expression, we observed increased expression of *Hnf4α* in both pH19-transfected and DEA-treated cells (Figure 4c). Together, these results suggest that H19 regulates promoter methylation and expression of *Hnf4α* in hepatic cells.

To determine whether this regulation also occurs *in vivo*, QMSP analysis was performed on genomic DNA isolated from livers of mouse fetuses with or without metformin exposure. On the basis of the results from Huh7 cells, we predicted hypomethylation of *Hnf4α* in metformin-exposed as compared to control livers. The three differentially methylated CpG sites (red highlighted, Figure 4a) in the conserved promoter region of mouse *Hnf4α* was identified by genome-wide methylation profiling (results to be published elsewhere). As shown in Figure 4d, there was clearly a decrease in *Hnf4α* promoter methylation in the liver of metformin-exposed *versus* control fetuses. The negative correlation between H19 expression and *Hnf4α* methylation (Figure 4e) further supports an *in vivo* regulation of *Hnf4α* methylation by H19. Collectively, these results suggest that increased *Hnf4α* expression is likely a result of decreased *Hnf4α* methylation, which is caused by an elevated expression of H19 by metformin in the fetal liver.

## Discussion

We show that in the mouse, metformin exposure throughout gestation reduces fetal bodyweight. It also downregulates *Insr* and upregulates H19 in the fetal liver, which induces hypomethylation and expression of *Hnf4α*, with subsequent activation of gluconeogenic genes. We also show that the H19-induced alteration of methylation in a conserved promoter region of *Hnf4α* is SAHH-dependent. We provide evidence that the H19 expression change is likely a direct effect of metformin on the fetal liver.

We believe that we have identified a potentially novel mechanism linking prenatal metformin exposure to adult onset of metabolic disorders in the mouse, which may have an important clinical implication for human patients. As the fetus has a continuous glucose supply from the mother *in utero*, hepatic gluconeogenesis normally is not activated until after birth. This has been attributed primarily to the low expression of rate-limiting gluconeogenic enzymes such as G6PC and PEPCK, and their regulatory transcriptional factors, including HNF4*α*.^29, 30^ However, premature activation of the gluconeogenic pathway, especially an increase in expression of *Hnf4α* and *Pck1/Pck2*, in fetuses exposed *in utero* to dexamethasone or to maternal HFD, has been associated with adulthood diabetic symptoms in both rodents and monkeys.^9, 31, 32^ This suggests that gluconeogenic gene activation before birth may be a common underlying mechanism for fetal programming of adult metabolic diseases. Indeed, a significant induction of *Hnf4α* has been seen in all three cases: gestational exposure to metformin (this report); to dexamethasone;^9^ or to maternal HFD.^31^ While previous studies reported an increase in expression of *Pck1* in maternal HFD^31, 32^ or *Pck2*^9^ in dexamethasone-^9^ exposed fetuses, we did not observe such an effect in metformin-exposed fetal liver (Figures 2d and e). Instead, we observed an increase in *G6pc* expression (Figure 2c). This discrepancy may arise from the different nature of exposure (metformin *versus* HFD *versus* dexamethasone).

Beyond its role as a gluconeogenesis regulator, HNF4*α* is indispensable for morphological and functional development of the liver. Loss of HNF4*α* not only blocks hepatocyte differentiation but also affects hepatic architecture as well. While some liver functions can be performed by all hepatocytes, other functions are limited to hepatocyte subsets. Proper lobular organization and the position of hepatocytes within the lobule are crucial for functional compartmentalization, a phenomenon called positional (or zonal) heterogeneity or metabolic zonation (reviewed in refs 25, 33, 34). It is thus conceivable that altered hepatic *Hnf4α* expression during fetal development as a result of gestational exposure to metformin, to dexamethasone, or to maternal HFD could produce profound effects on liver function, and hence whole-body energy homeostasis later in life.

A previous study showed that treating dams on HFD before and during gestation with metformin throughout pregnancy protected offspring from developing glucose intolerance.^19^ However, this result was confounded by the fact that the study used a mouse model that did not separate effects of maternal diabetes from HFD, given that HFD in the absence of maternal diabetes has been shown to induce T2D in the adult offspring.^31, 32, 35^ Therefore, this mouse model was not suitable for studying metformin effects on offspring of mothers who were treated with metformin for T2D or GDM during pregnancy. While the regular-diet mouse model we used in this report does not mimic the pregnant human patients treated with metformin for T2D or GDM, it does provide a cautionary note that metformin has the potential of directly impacting the fetus by altering hepatic development and function, irrespective of the conditions of the mother. Thus, using metformin-like drugs that do not pass the placenta may remove the potentially negative effects of metformin on the offspring.

It was initially unexpected to observe that metformin upregulates *H19* expression both in mouse fetal livers (Figure 2a) and in human Huh7 hepatoma cells (Figure 3d), given that metformin inhibits *H19* expression in human endometrial and ovarian cancer cells.^16, 36^ Metformin has also long been known to repress gluconeogenesis in the adult liver.^37^ However, this apparent contradiction could be explained by cell/tissue-dependent and developmental stage-dependent effects of metformin. It is intriguing to observe a decreased expression of *Insr* in liver of metformin-exposed fetuses (Figure 2f), a likely indirect effect of metformin, as metformin did not alter *Insr* expression in Huh7 cells (data not shown). Insulin signaling has been implicated in liver enzymic differentiation in the fetus, as fetal hyperinsulinemia was associated with reduced activity of G6PC and PEPCK (reviewed in ref. 33). Although speculative, the decreased *Insr* expression in the fetal liver might contribute to increased expression of gluconeogenic genes. Finally, the mechanism by which *H19* is upregulated and *Insr* is downregulated by metformin in the fetal liver remains to be investigated.

**Figure 5:**
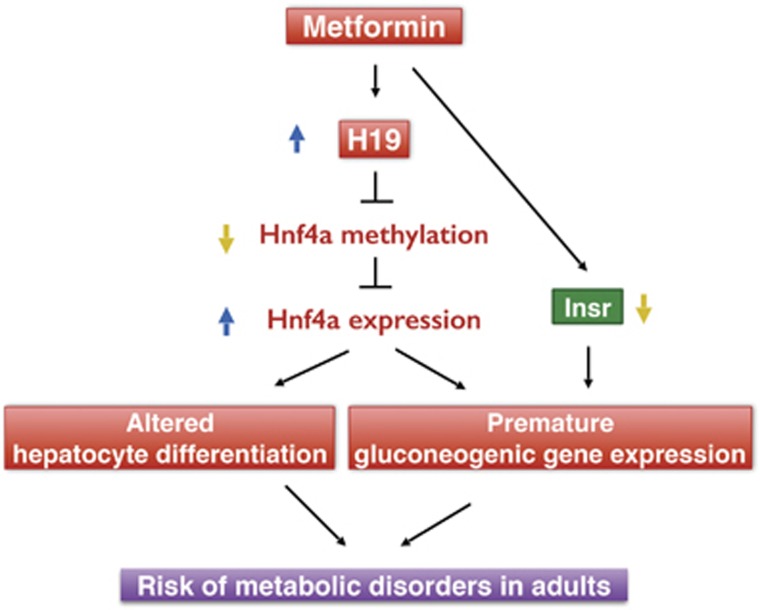
A proposed model

In summary, we propose a model illustrated in Figure 5. In this model, gestational metformin exposure directly upregulates *H19* and indirectly downregulates *Insr* in the fetal liver. H19 acts to decrease *Hnf4α* methylation, thereby increasing *Hnf4α* expression, which drives premature gluconeogenic gene expression; decreased insulin signaling also contributes to this process. Increased *Hnf4α* expression also alters hepatocyte differentiation. Together, they increase the risk of metabolic disorders in adult life.

## Materials and methods

### Materials

Antibodies for G6PC (Abcam, Cambridge, MA, USA; ab83690; used at a dilution of 1/500), HNF4*α* (Abcam, ab181604; used at a dilution of 1/1000), PEPCK (Abcam, ab70358; used at a dilution of 1/1000), and beta-actin (ACTB; Cell Signaling, 4967; used at a dilution of 1/5000) were purchased. Plasmids expressing human H19 (pH19) and empty vector (Vec) were previously described.^27^ Metformin (ENZO Life Sciences International Inc., Uniondale, NY, USA; ALX-270-432-G005) and DEA (Santa Cruz, Dallas, TX, USA; sc-207632) were purchased. DEA was used at a final concentration of 20 μM.

### Animals

All animal work was approved by the Yale University Institutional Animal Care and Use Committee. CD-1 mice were obtained from Charles River Laboratories (Wilmington, MA, USA). Mice were housed at 22–24 °C with a 12 h light/12 h dark cycle with standard chow (Purina Chow; Purina Mills, Richmond, IN, USA) and water provided *ad libitum*. Eight-week-old female mice were mated to male mice. The morning of the detection of a vaginal plug was designated as E0.5. Pregnant mice were then housed individually and metformin or control (tap water) was administrated orally at 250 mg/kg from E0.5 through E18. On gestational day 18.5 (E18.5), pregnant dams were fasted for 6 h and then killed by standard carbon dioxide inhalation before they underwent cesarean delivery to collect tissues from dams and offspring. Fetal weights were recorded. Tissue samples were snap-frozen in liquid nitrogen and stored at −80 °C for further analysis. Blood samples were collected by cardiac puncture from the dams in terminal anesthesia with ISOTHESIA (isoflurane, Henry Schein Animal Health, Dublin, OH, USA). Serum samples were used for blood glucose measurement using Bayer’s Breeze2 Blood Glucose Monitoring System (Bayer, Leverkusen, Germany; 440C) according to the manufacturer’s instructions.

### Cell culture and transfection

Huh7 cells (ThermoFisher Scientific LLC, Suwance, GA, USA; NC0377393) were authenticated and were free from mycoplasma contamination. The cells were cultured in low-glucose DMEM (Gibco, Grand Island, NY, USA; 11885-085) supplemented with 10% fetal bovine serum, heat-inactivated, and 1% penicillin/streptomycin. Cells were transfected as previously described.^15, 16^ RNA, genomic DNA, and protein were extracted and analyzed at the indicated time points following transfection.

### Metformin treatment of Huh7 cells

Huh7 cells growing in 24-well plates were incubated with metformin in growth medium at the indicated concentrations. RNAs were extracted 48 h later and analyzed by RT-qPCR.

### RNA extraction and RT-qPCR

Total RNA was extracted from fetal liver tissue samples or from Huh7 cells using PureLink RNA Mini Kit (Ambion, Grand Island, NY, USA; 12183018A). cDNA was synthesized using PrimeScript RT Reagent Kit (Takara, Mountain View, CA, USA; RR037A) in a 20 *μ*l reaction containing 100–200 ng of total RNA. RT-qPCR was performed as previously described.^15, 16^ Gene expression levels were normalized against house-keeping genes *Hprt1* and *Rpl0*. The real-time PCR primers are listed below.

Mouse H19 forward: 5′-CCTCAAGATGAAAGAAATGGTGCTA-3′

Mouse H19 reverse: 5′-TCAGAACGAGACGGACTTAAAGAA-3′

Mouse Hnf4*α* forward: 5′-TCTTCTTTGATCCAGATGCC-3′

Mouse Hnf4*α* reverse: 5′-GGTCGTTGATGTAATCCTCC-3′

Mouse Insr forward: 5′-CCCCACCCTTTGAGTCTGAT-3′

Mouse Insr reverse: 5′-CTGTCACATTCCCCACCTCT-3′

Mouse G6pc forward: 5′-ATCCGGGGCATCTACAATG-3′

Mouse G6pc reverse: 5′-TGGCAAAGGGTGTAGTGTCA-3′

Mouse Pck1 forward: 5′-TGTTTACTGGGAAGGCATCG-3′

Mouse Pck1 reverse: 5′-AGGTCTACGGCCACCAAAG-3′

Mouse Pck2 forward: 5′-CCCTATCACAAGGCAAGAGA-3′

Mouse Pck2 reverse: 5′-CCACTTCCCCTGTCCTATTT-3′

Mouse Hprt1 forward: 5′-CAGTCCCAGCGTCGTGATTA-3′

Mouse Hprt1 reverse: 5′-GGCCTCCCATCTCCTTCATG-3′

Mouse Rpl0 forward: 5′-GATGGGCAACTGTACCTGACTG-3′

Mouse Rpl0 reverse: 5′-CTGGGCTCCTCTTGGAATG-3′

Mouse Igf2 forward: 5′-GCTTGTTGACACGCTTCAGTTTG-3′

Mouse Igf2 reverse: 5′-GTTGGCACGGCTTGAAGGC-3′

Human H19 forward: 5′-ACTCAGGAATCGGCTCTGGAA-3′

Human H19 reverse: 5′-CTGCTGTTCCGATGGTGTCTT-3′

Human Hnf4*α* forward: 5′-CAGAATGAGCGGGACCGGATC-3′

Human Hnf4*α* reverse: 5′-CAGCAGCTGCTCCTTCATGGAC-3′

Human Igf2 forward: 5′-CCGAAACAGGCTACTCTCCT-3′

Human Igf2 reverse: 5′-AGGGTGTTTAAAGCCAATCG-3′

Human G6pc forward: 5′-CCTCAGGAATGCCTTCTACG-3′

Human G6pc reverse: 5′-TCTCCAATCACAGCTACCCA-3′

Human Pck1 forward: 5′-GGTTCCCAGGGTGCATGAAA-3′

Human Pck1 reverse: 5′-CACGTAGGGTGAATCCGTCAG-3′

Human Prt1 forward: 5′-GACCAGTCAACAGGGGACAT-3′

Human Prt1 reverse: 5′-CCTGACCAAGGAAAGCAAAG-3′

Human Rpl0 forward: 5′-GGCGACCTGGAAGTCCAACT-3′

Human Rpl0 reverse: 5′-CCATCAGCACCACAGCCTTC-3′

### Western blot analysis

Protein levels were determined by SDS-polyacrylamide gel electrophoresis followed by blotting against their specific antibodies as previously described.^15, 16^

### Glucose output assay

This was performed using Amplex Red Glucose/Glucose Oxidase Assay Kit (A22189, Molecular Probes, Invitrogen, Carlsbad, CA, USA), according to the manufacturer’s instructions. Briefly, Huh7 cells were transfected with pH19 or Vec in a 24-well plate. Forty-eight hours later, culture medium was replaced with glucose-free DMEM (Gibco; 11966-025) for 2 h and then incubated in 120 *μ*l glucose production media (glucose-free DMEM, 20 mM sodium lactate, 2 mM sodium pyruvate, and 0.5% BSA) for 4 h. Subsequently, 50 *μ*l of supernatant was used for measurement of glucose concentration, which was normalized to total protein content of cells.

### Quantitative methylation-specific PCR

Genomic DNA was extracted from fetal liver tissue samples or from Huh7 cells in one well of 24-well plates using Quick-gDNA MicroPrep (Zymo Research Corporation, Irvine, CA, USA; D3021) according to the manufacturer’s instructions. For bisulfite treatment, 400–500 ng of DNA was used for each column using EZ DNA Methylation-Gold Kit (Zymo, D5006). A volume of 100 *μ*l of elution buffer was used to elute DNA from each column. RT-qPCR was performed in a 15 *μ*l reaction containing 5 *μ*l of the eluant using iQSYBRGreen in a Bio-Rad (Hercules, CA, USA) iCycler. The PCR primers for methylated DNA were used at a final concentration of 0.3 *μ*M in each PCR reaction. PCR was performed by initial denaturation at 95 °C for 5 min, followed by 40 cycles of 30 s at 95 °C, 30 s at 60 °C, and 30 s at 72 °C. Ct values of each sample were used in the post-PCR data analysis. Albumin DNA was used as loading controls for QMSP normalization. The primers used for QMSP are listed below.

Human Hnf4*α* methylated forward: 5′-TTTAATTTTAGAGTGTAGGATTAGGATTCG-3′

Human Hnf4*α* methylated reverse: 5′-TCTTCTAATCACCCAAAATAAATAAATACG-3′

Human albumin forward: 5′-GTATGCCTGAGCCCCAAAGT-3′

Human albumin reverse: 5′-CCTTGGGCTTGTGTTTCACG-3′

Mouse Hnf4*α* methylated forward: 5′-GATTAGAAGAATTAATAAGATAATCGGGC-3′

Mouse Hnf4*α* methylated reverse: 5′-AAACAAAAACCCACACACAACAAC-3′

Mouse albumin forward: 5′-GTGAGAATTGTAGAGCAGTGCTGTC-3′

Mouse albumin reverse: 5′-ACATTGCTCAGCACAGATCCAC-3′

### Statistical analysis

Statistical analyses were performed using the Statistical Package for the Social Science (SPSS) computer software version 17.0 (IBM SPSS Statistics, Chicago, IL, USA). *In vivo* gene expression data are presented as median and interquartile range and analyzed using Mann–Whitney *U*-test. Spearman correlations were performed for gene co-expression analyses. *In vitro* data are presented as mean±S.D. and analyzed using two-tailed Student’s *t-*test. Figures were constructed using Prism 6 version 6.0f (GraphPad Software, Inc., La Jolla, CA, USA). *P-*values at 0.05 or smaller (two-sided) were considered statistically significant.
