# Correction: H19 lncRNA alters methylation and expression of *Hnf4α* in the liver of metformin-exposed fetuses

**DOI:** 10.1038/s41419-019-1812-x

**Published:** 2019-08-07

**Authors:** Jie Deng, Martin Mueller, Tingting Geng, Yuanyuan Shen, Ya Liu, Peng Hou, Ramanaiah Mamillapalli, Hugh S. Taylor, Michael Paidas, Yingqun Huang

**Affiliations:** 1 Department of Obstetrics, Gynecology, & Reproductive Sciences, Yale University School of Medicine, New Haven, CT 06510 USA; 2 Department of Obstetrics and Gynecology, University Hospital, Bern, Switzerland; 3 Department of Endocrinology, First Affiliated Hospital of Xi’an Jiaotong University School of Medicine, Xi’an, 710061 China; 4 Department of Endocrine and Breast Surgery, The First Affiliated Hospital of Chongqing Medical University, Chongqing, 400016 China; 5 Department of Veterinary Medicine, College of Animal Science and Technology, Anhui Agricultural University, Anhui, 230036 China

**Keywords:** Cell biology, Catalysis, Acute coronary syndromes, Anthropology

**Correction to:** Cell Death & Disease

10.1038/cddis.2017.392 published online 7 December 2017

Since publication of this article, Dr Ramanaiah Mamillapalli reported that his last name had published incorrectly as Ramillapalli. The publisher apologizes to the authors and to readers for this error, which has not been fixed in the original article.

